# Influence of perceived stress on professional identity among nursing students: a chain mediating role of self-control and self-directed learning ability

**DOI:** 10.3389/fmed.2024.1429014

**Published:** 2024-11-12

**Authors:** Xin Zhao, Wen-Kai Zheng, Xiu-Huan Wang, Jiao Fang, Wen-Jin Chen, Na Li, Hai-Tao Wen, Xiu-Juan Feng, Mei-Fang Wang, Chun-Ni Heng, Wei-Na Cao

**Affiliations:** ^1^Shaanxi Provincial People's Hospital, Xi'an, China; ^2^School of Basic Medicine, Inner Mongolia Medical University, Hohhot, China; ^3^Peking University HuiLongGuan Clinical Medical School, Beijing, China; ^4^College of Nursing and Rehabilitation, Xi'an Jiaotong University City College, Xi'an, China; ^5^Tangdu Hospital, Fourth Military Medical University, Xian, Shaanxi, China

**Keywords:** perceived stress, nursing student, professional identity, self-control, self-directed learning ability

## Abstract

**Background:**

A positive professional identity is key for nursing students in determining career direction and predicting future engagement in the profession. Despite its complexity and susceptibility to various influences, the factors shaping nursing students' professional identity remain poorly understood.

**Objectives:**

This study aims to investigate how perceived stress can directly and indirectly influence professional identity among nursing students, with self-control and self-directed learning ability as mediators.

**Materials and methods:**

A cross-sectional survey was conducted from October to December 2023, collecting data from 675 nursing students across five tertiary hospitals in Xi'an, Shaanxi Province, China. The survey captured detailed data on sociodemographic characteristics, perceived stress, self-control, self-directed learning ability, and professional identity among the participants. Descriptive analysis and correlation matrices were used to analyze participant characteristics and assess bivariate correlations. The mediation model was analyzed using the PROCESS macro for SPSS.

**Results:**

Perceived stress showed a direct and negative influence on professional identity among nursing students; self-control was shown to play a mediating role between perceived stress and professional identity; self-directed learning ability was shown to play a mediating role between perceived stress and professional identity; and self-control and self-directed learning ability were shown to play a chain mediating role between perceived stress and professional identity.

**Conclusion:**

Self-control and self-directed learning ability have a chain mediating role in between perceived stress and professional identity among nursing students. It suggests that nursing managers and educators can improve the self-control and self-directed learning ability of nursing students to mitigate the negative impact of perceived stress on professional identity.

## Introduction

Professional identity is an extremely important professional psychological quality for clinical nurses, which not only affects their commitment and effort toward their profession, but also has profound effects on job satisfaction, work motivation, and career path choices (1, 2). For nursing interns (referred to as nursing students), a strong professional identity is the starting point and core for them to determine their career direction, and it is also a key factor in predicting their future engagement in the nursing profession (3, 4). Previous studies have found that positive professional identity among nursing students has a protective effect on their choice to continue their career path in nursing, which is of great significance for the stability of the nursing workforce (5). However, Chen et al. found that the overall level of professional identity among nursing students in China is moderate, and stress is one of the main factors leading to a reduction in their sense of professional identity (6). According to the stress-adaptation theory, individuals, when faced with external stressors, exhibit corresponding emotional and cognitive responses, which in turn affect their adaptive capacity (7). This theory posits that an individual's perception of stress influences their emotional reactions, such as anxiety and tension, subsequently impacting their ability to adapt to and identify with their environment (7). In the context of nursing interns, stressors during the internship may include heavy workloads, unfamiliarity with clinical procedures, and uncertainties about future career development. These perceived stressors may affect nursing students' professional identity through emotional responses, diminishing their positive perception and sense of belonging to the nursing profession. Zhang et al. found that perceived stress is a significant negative predictor of professional identity (8). The clinical internship period, as the final stage of nursing education, is an important period for nursing students to integrate theory and practice, and it is also a critical period for the formation of professional identity (9). Improving and enhancing nursing students' professional identity has always been the goal of nursing educators and managers. This study seeks to explore the relationship between perceived stress and professional identity. The relationship between perceived stress and professional identity is multifaceted, involving both direct and indirect effects. The self-determination theory emphasizes the critical role of motivation, autonomy, and competence in regulating individual behavior (10). Self-control and self-directed learning ability can be regarded as vital components of intrinsic motivation. These abilities not only assist nursing students in effectively coping with the pressures of their academic and internship experiences but also foster the development of their professional identity within the nursing field (11–13). So, this study investigates the indirect effects of perceived stress on professional identity, with self-control and self-directed learning acting as the mediating variables.

Self-control is defined as the capacity to resist external temptations while fulfilling internal desires to achieve personal goals (14). Perceived stress exerts a detrimental effect on self-control, often accompanied by negative emotions that disrupt an individual's focus and cognitive functioning, thereby reducing the overall capacity for self-regulation (15, 16). Nursing students possessing high levels of self-control are better equipped to resist short-term temptations and distractions, thereby sustaining long-term focus and commitment to their profession. These students demonstrate the capability to formulate and adhere to career plans, gradually progressing in their professional development, which subsequently enhances their sense of professional identity. Consequently, we infer that elevated levels of self-control facilitate the cultivation of a positive professional identity and also mitigate the adverse effects of perceived stress on professional identity.

Self-directed learning ability is defined as an individual's capacity for self-management, self-regulation, and independent decision-making throughout the learning process (17, 18). Perceived stress exerts a negative influence on self-directed learning ability (19). Nursing students demonstrating high self-directed learning ability generally possess a heightened sense of professional identity (13). Thus, self-directed learning ability exerts a positive influence on professional identity. Therefore, we infer that self-directed learning ability may alleviate the negative influence of perceived stress on professional identity.

**Figure 1:**
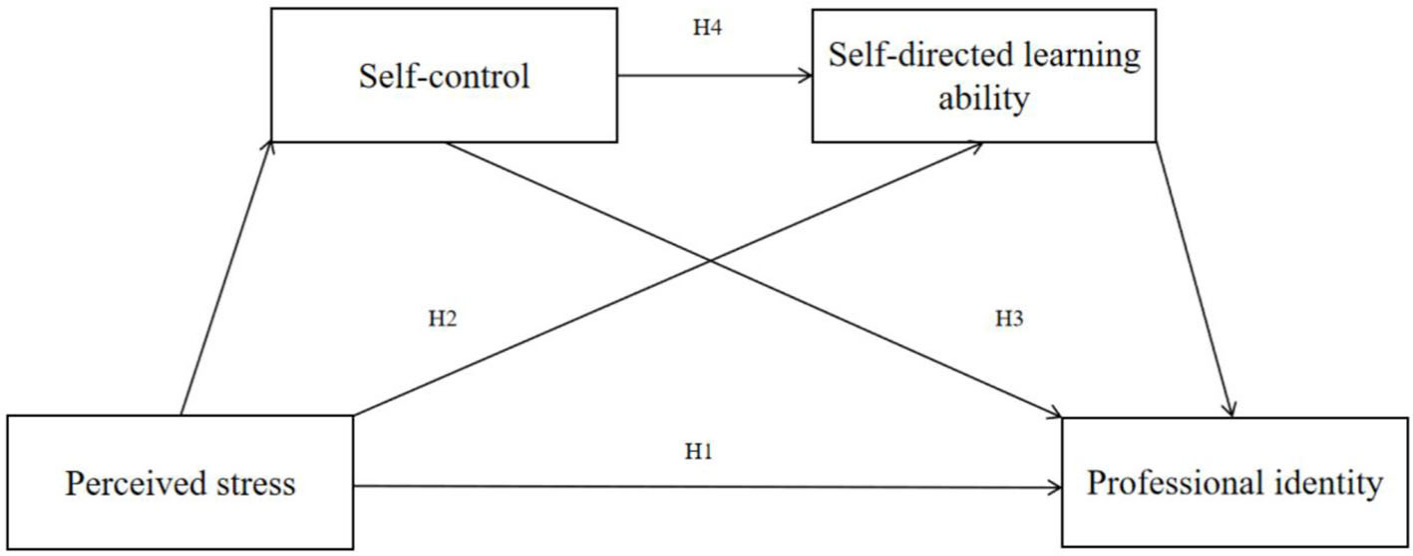
Hypothetical conceptual model.

Although the influence of perceived stress on professional identity is substantiated by existing literature, research exploring the mediating effects of self-control and self-directed learning ability remains relatively limited, especially studies concentrating on chain mediation effects. To further elucidate the mechanisms through which perceived stress influences professional identity, this study focuses on nursing students and employs self-control and self-directed learning ability as mediating variables, thereby constructing a chain mediation model involving the four variables. This model not only assesses the direct influence of perceived stress on professional identity but also investigates the indirect effects of self-control and self-directed learning ability, enriching the influence path of perceived stress on professional identity. This study provides a basis for nursing educators and administrators to improve teaching strategies and curriculum design, aiding students in better managing stress during their studies and internships while enhancing their sense of professional identity. The research model is depicted in Figure 1, and the research questions (RQ) addressed were follows:

RQ1: How does perceived stress influence professional identity?
RQ2: How does self-control mediate the relationship between perceived stress and professional identity?
RQ3: How does self-directed learning ability mediate the relationship between perceived stress and professional identity?
RQ4: How do self-control and self-directed learning ability play a chain mediating role between perceived stress and professional identity?

## Hypotheses development

### Influence of perceived stress on professional identity

Perceived stress refers to the individual's assessment of the degree to which a stimulus event produces stress on oneself, which is a form of perceived stress (20). According to the stress-adaptation theory, perceived stress can influence an individual's emotional and cognitive responses, thereby affecting their adaptability (7). For nursing students, if they perceive the difficulties and challenges encountered during their internship as stressors and feel threatened by them, it may lead to negative emotions and cognitive responses, thus reducing their sense of identification with the nursing profession. Although there has been initial evidence from abroad confirming the relationship between nursing students' perceived stress and professional identity, there is still a relative lack of relevant research in China. Therefore, the following hypothesis is proposed:

H1: Perceived stress negatively influences professional identity.

### Mediating role of self-control

Self-control refers to an individual's ability to effectively regulate and manage their thoughts, emotions, and behaviors in order to achieve long-term goals (14). Career development theory emphasizes that at different stages of individual growth, they need to complete specific tasks to promote psychological and social development (21). The theory suggests that individuals use their self-control abilities to manage their behaviors and emotions, set career goals, take appropriate actions, and thus better adapt to the needs of career development and establish professional identity (21). Zhang et al. found that a negative correlation between perceived stress and self-control among college students (16). Zheng et al. found that perceived stress serves as a negative predictor of self-control abilities among nursing undergraduates, with elevated stress levels frequently associated with negative emotions such as anxiety, tension, and anger (15). Negative emotions have the potential to disrupt an individual's rational thinking and decision-making capabilities, rendering them susceptible to impulsive behaviors that may compromise self-control (15). Considering that perceived stress has a negative predictive effect on self-control, whereas self-control exerts a positive predictive influence on professional identity, this study proposes the following hypothesis:

H2: Self-control plays a mediating role between perceived stress and professional identity.

### Mediating role of self-directed learning ability

Self-directed learning ability refers to an individual's capacity for self-management, self-regulation, and autonomous decision-making during the learning process (17, 18). Studies have found that perceived stress negatively predicts self-directed learning ability (19, 22). Dynamic theory holds that the behavior of individuals is the result of the interaction between the environment and the individual, emphasizing the adaptability and variability of behavior (23). The theory indicates that stress negatively impacts the learning process, primarily in two ways: first, it induces emotional tension and anxiety, leading to decreased attention, which affect learning outcomes and self-directed learning ability; second, it triggers negative emotions and diminishes learning motivation, resulting in a lack of initiative and enthusiasm, thereby further reducing self-directed learning ability (19, 22, 23). Studies have found that self-directed learning ability positively predicts professional identity (12, 13). Nursing students with higher self-directed learning ability are more likely to clarify their career goals and directions, independently develop study plans and action plans, consciously select learning resources and opportunities related to the nursing profession, and clear career goals contribute to shaping their professional identity. Considering that perceived stress has a negative predictive effect on self-directed learning ability, whereas self-directed learning ability exhibits a positive predictive influence on professional identity, this study articulates the following hypothesis:

H3: Self-directed learning ability plays a mediating role between perceived stress and professional identity.

### Chain mediating role of self-control and self-directed learning ability

Research indicates that perceived stress negatively predicts self-control (16), while self-control positively predicts self-directed learning ability (24). A comprehensive learning process necessitates that individuals regulate their learning behaviors through mechanisms such as self-monitoring, strategic planning, execution, and reflective evaluation (18, 25). Self-control is pivotal in enabling individuals to sustain focus and patience, particularly when confronting complex learning tasks (26). Self-control facilitates the improvement of self-directed learning through two primary mechanisms. Firstly, individuals exhibiting higher self-control are more likely to maintain intrinsic motivation and autonomy, thereby significantly enhancing their self-directed learning capacities. Secondly, individuals with superior self-control demonstrate an enhanced ability to maintain focus throughout the learning process and are less susceptible to interruptions from external distractions, ultimately optimizing learning efficiency (16, 18, 25, 26). According to self-determination theory (10), an individual's self-directed learning ability depends on their intrinsic motivation and sense of autonomy in learning activities. Generally, individuals with higher self-control abilities are able to autonomously choose learning goals, develop learning plans, and make choices regarding learning strategies, which is often associated with higher levels of self-directed learning ability. Chen et al. found that self-control positively predicts self-directed learning ability (25). Previous studies have consistently shown that self-directed learning ability positively predicts professional identity (12, 13, 27). Considering that perceived stress negatively predicts self-control, self-control positively influences self-directed learning ability, and self-directed learning ability positively affects professional identity, this study posits the following hypothesis:

H4: Self-control and self-directed learning ability play a chain mediators between perceived stress and professional identity.

## Materials and methods

### Design and participants

A cross-sectional study was conducted in October to November 2023, using a convenience sampling method to select nursing students from five tertiary grade A hospitals as the study subjects in Shaanxi Province, China. Inclusion criteria: (1) Full-time four-year undergraduate nursing students; (2) Currently in the clinical internship period; (3) Internship duration ≥ 3 months; (4) Informed consent and voluntary completion of the questionnaire. Exclusion criteria: Students who have not completed the questionnaire survey for various reasons. The sample size was calculated with the formula *N* = 4Uα^2^S^2^/δ^2^ (28). Based on preliminary experimental results, *S* = 0.60. The allowable error δ is set to 0.1, and α is set to 0.05. Therefore, *N* = 4 × 1.96^2^ × 0.60^2^/0.1^2^≈553. Considering a 15% invalid questionnaire rate, the minimum sample size set to 636. A total of 675 nursing students were included in this study-−39 male students and 636 female students.

### Procedure

After establishing contact with the nursing education team leaders of five tertiary grade A hospitals in Xi'an, Shaanxi Province, the researchers provided a detailed introduction of the purpose and content of the study, and obtained their consent. Upon receiving consent, the researchers sent the pre-designed questionnaire star link to the hospital administrators via WeChat, who then forwarded it to the nursing students. Participants anonymously completed the questionnaire. This study utilized the Questionnaire Star platform to prevent duplicate responses, by setting a limit of one response per IP address. Data collection lasted from October to November 2023. A total of 705 questionnaires were collected for this study. Of these, 30 questionnaires were excluded: nine due to missing responses, 13 due to high response homogeneity across all items, and eight for having completion times of <5 min. Consequently, 675 valid questionnaires were retained for data analysis.

### Measurements

#### Sociodemographic characterization

Sociodemographic characterization involved gender (male, female), one-child (yes, no), type of residence (downtown, suburb, and rural areas), and family finances (low income, middle income, and high income).

#### Perceived Stress Scale

The PSS developed by Yang and Huang was used to measure perceived stress levels of nursing students (29). The PSS consists of two dimensions: perceived tension (seven items) and perceived lack of control (seven items), including 14 items total (Example items: 1. Feeling upset about something unexpected. 2. Feeling unable to control the important things in your life. 3. Feeling nervous and stressful.). Each item was rated on a 5-point Likert scale, from 1 (never) to 5 (always). The PSS is scored on a range of 14–70, with higher scores indicating a higher level of perceived stress. Studies have shown that the PSS has good reliability and validity for this Chinese population (29, 30). The Cronbach alpha coefficient of the scale in this study was 0.768.

#### Self-Control Scale

The SCS developed by Tan and Guo was used to measure self-control ability of nursing students (31). The SCS consists of five dimensions: impulse control (six items), health habits (three items), resisting temptation (four items), work concentration (three items), and entertainment moderation (three items), including 19 items total (Example items: 1. I can resist temptation very well. 2. It is difficult for me to change my bad habits. 3. I am lazy.). Each item was rated on a 5-point Likert scale, from 1 (completely does not match) to 5 (completely matches). The SCS is scored on a range of 19–95, with higher scores indicating better self-control ability. The SCS has been widely applied among Chinese population and found to have good reliability and validity (31, 32). The Cronbach alpha coefficient of the scale in this study was 0.889.

#### Self-Directed Learning Ability Scale

The SLAS developed by Lin and Jiang was used to measure self-directed learning ability of nursing students (33). The SLAS consists of three dimensions: self-management ability (10 items), information ability (11 items), and learning cooperation ability (seven items), including 28 items total (Example items: 1. Honestly, it's challenging to gauge my understanding without a test score. 2. Honestly, I'm unsure which part of the knowledge is problematic. 3. I will set my next learning goals based on the challenges I encountered during my studies.). Each item is scored on a 5-point scale. For items 1 to 21, responses range from “A = completely inconsistent” to “E = completely consistent,” scored from 1 to 5 points, respectively. For items 22–28, responses from A to E are scored 5 to 1 points, respectively. Items 1, 2, 5, 10, 11, 13, and 27 are reverse-scored. The total score ranges from 28 to 140 points, with higher scores indicating greater self-directed learning ability. The SLAS has been extensively used among Chinese nursing students and has demonstrated strong reliability and validity (33, 34). The Cronbach alpha coefficient of the scale in this study was 0.828.

#### Professional Identity Questionnaire

The PIQ developed by Hao was used to measure professional identity levels of nursing students (35). The PIQ consists of five dimensions: professional self-concept (six items), retention benefits and turnover risks (four items), social comparison and self-reflection (three items), autonomy in career choices (two items), and social persuasion (two items), making a total of 17 items (Example items: 1. I take great pride in being a nurse. 2. I am deeply interested in learning about the career journeys of successful nursing professionals. 3. I am eager to engage with experienced professionals in the nursing field.). Each item was rated on a 5-point Likert scale, from 1 (strongly disagree) to 5 (strongly agree). The PIQ is scored on a range of 17–85, with higher scores indicating a higher level of professional identity. The PIQ has been widely applied among Chinese nursing students and has showed strong reliability and validity (35, 36). The Cronbach alpha coefficient of the scale in this study was 0.943.

### Statistical methods

Statistical analysis was performed using SPSS 27.0. The data for this study was collected through the questionnaire star platform using a self-report method, which may lead to common method bias. Harman's single-factor method was used to test for common method bias. Frequency was used to describe categorical data, while mean and standard deviation were used to describe continuous data. Independent samples *t*-test was conducted to analyze the differences in perceived stress, self-control, self-directed learning ability, and professional identity between male and female nursing students. Pearson correlation analysis was used to examine the relationships between variables (perceived stress, self-control, self-directed learning ability, and professional identity). The mediating effect of self-control and self-directed learning ability between perceived stress and professional identity was examined using Model 6 in the SPSS Process macro program for mediation model construction and testing, and the mediation effect was tested by bootstrap with 5, 000 repeated samples (37). This method is based on the least squares regression and the bootstrap method. Statistical significance was determined at a *p*-value of <0.05.

### Ethics statement

The present study was approved by the Ethics Committee of Tangdu Hospital (Approval ID: TDLL-202210-17) and complied with the Declaration of Helsinki. The purpose of the study was explained to all participants before the survey was conducted and informed consent was obtained.

## Results

### Common method bias test

This study tested for common method bias using Confirmatory Factor Analysis (CFA) (38). In the CFA, we constructed a single latent common factor model, assuming that this factor could explain the variance of all measurement items. However, the analysis results showed that the single-factor model had poor fit indices and did not meet acceptable fit standards. This indicates that the single common factor could not effectively explain the variance of the measurement items, suggesting that there is no significant common method bias in the data (38).

### Sociodemographic characterization

Table 1 presents the demographic data and scores of professional identity. Among the 675 nursing students surveyed, 94.22% were female, 21.04% were only children, 55.26% came from rural areas, and 82.07% of students self-rated their family's economic income as moderate.

**Table 1:** Sociodemographic characteristics and comparison of professional identity among nursing students.

**Variables**	**Number (*N*)**	**Percentage (%)**	**Professional Identity**		***t*/*F***	***P***
			**Mean**	**SD**		
Gender					1.455	0.146
Male	39	5.78	59.03	12.17		
Female	636	94.22	56.30	11.31		
One-child					0.714	0.475
Yes	142	21.04	57.06	13.57		
No	533	78.96	56.30	10.71		
Type of residence					0.271	0.763
Downtown	155	22.96	55.90	13.60		
Suburb	147	21.78	56.42	12.05		
Rural areas	373	55.26	56.70	10.02		
Family finances					2.790	0.062
Low income	107	15.85	54.54	11.82		
Middle income	554	82.07	56.92	11.08		
High income	14	2.08	52.64	16.64		

There were no statistically significant differences among nursing students of different genders, one-child, type of residence, and family finances in professional identity level (*p* > 0.05), as shown in Table 1.

### Association between perceived stress, self-control, self-directed learning ability, and professional identity

Table 2 shows that the average scores of perceived stress, self-control, self-directed learning ability, and professional identity among nursing students were (39.70 ± 6.20), (62.58 ± 10.41), (87.85 ± 9.56), and (56.46 ± 11.37), respectively. Perceived stress among nursing students was negatively correlated with self-control (*r* = −0.546, *p* < 0.001), self-directed learning ability (*r* = −0.458, *p* < 0.001), and professional identity (*r* = −0.413, *p* < 0.001); Self-control was positively correlated with self-directed learning ability (*r* = 0.498, *p* < 0.001) and professional identity (*r* = 0.376, *p* < 0.001); Self-directed learning ability was positively correlated with professional identity (*r* = 0.448, *p* < 0.001).

**Table 2:** Correlational analysis of perceived stress, self-control, self-directed learning, and professional identity.

**Variables**	**1**	**2**	**3**	**4**	**Cronbach's alpha**
1. Perceived stress	1				0.768
2. Self-control	−0.546^***^	1			0.889
3. Self-directed learning ability	−0.458^***^	0.498^***^	1		0.828
4. Professional identity	−0.413^***^	0.376^***^	0.448^***^	1	0.943
Mean	39.70	62.58	87.85	56.46	–
Standard deviation	6.20	10.41	9.56	11.37	–

^***^p < 0.001.

### Mediation analysis of self-control and self-directed learning ability between perceived stress and professional identity

Gender, one-child status, type of residence, and family finances were included as covariates in the analysis. Regression analysis showed that perceived stress negatively predicted self-control (β = −0.544, *p* < 0.001), self-directed learning ability (β = −0.263, *p* < 0.001), and professional identity (β = −0.205, *p* < 0.001). Self-control positively predicted self-directed learning ability (β = 0.350, *p* < 0.001) and professional identity (β = 0.102, *p* < 0.01). Self-directed learning ability had a positive predictive effect on professional identity (β = 0.275, *p* < 0.001) (see Table 3).

**Table 3:** Mediating effect of self-control and self-directed learning ability in the relationship between perceived stress and professional identity.

**Regression model**		**Overall fit index**			**Significance of regression coefficients**	
**Dependent variable**	**Independent variable**	***R***	*R* ^2^	***F***	β	***t***
Self-control		0.548	0.300	57.267		
	Perceived stress				−0.544	−16.727^***^
	Gender				−0.109	−0.745
	One-child				−0.016	−0.190
	Type of residence				0.037	0.848
	Family finances				−0.001	−0.002
Self-directed learning ability		0.546	0.298	47.355		
	Self-control				0.350	9.094^***^
	Perceived stress				−0.263	−6.837^***^
	Gender				−0.108	−0.743
	One-child				−0.017	−0.200
	Type of residence				0.011	0.260
	Family finances				−0.012	−0.134
Professional identity		0.516	0.266	34.517		
	Self-directed learning ability				0.275	7.349^***^
	Self-control				0.102	2.582^**^
	Perceived stress				−0.205	−5.323^***^
	Gender				−0.170	−1.211
	One-child				−0.056	−0.675
	Type of residence				0.042	1.009
	Family finances				0.007	0.081
Professional identity		0.419	0.175	28.439		
	Perceived stress				−0.386	−11.635^***^
	Gender				−0.222	−1.489
	One-child				−0.064	−0.729
	Type of residence				0.052	1.188
	Family finances				0.004	0.040

^**^p < 0.01, ^***^p < 0.001.

**Figure 2:**
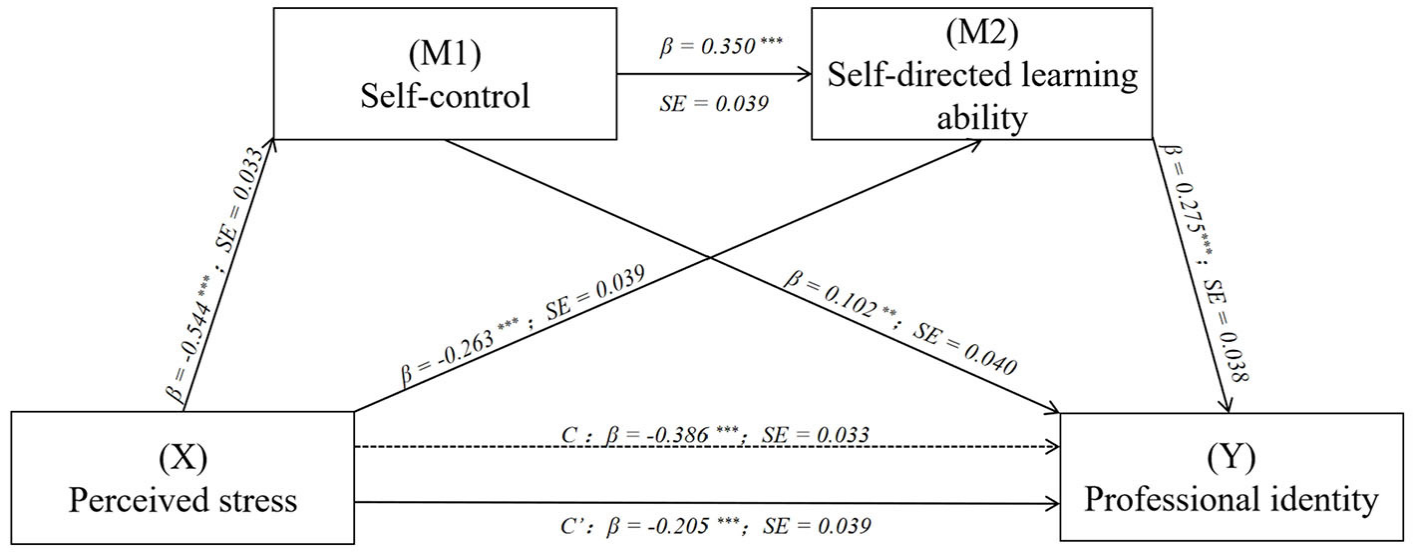
The mediation of self-control and self-directed learning ability in the relationship between perceived stress and professional identity with standardized bete values and standard error. **p < 0.01, ***p < 0.001.

Mediation analysis revealed significant mediating effects of self-control and self-directed learning ability, with a mediation effect value of −0.181, accounting for 46.89% of the total effect. Specifically, the mediation effects were produced through three mediation chains: (1) Perceived stress → Self-control → Professional identity [effect value = −0.056, accounting for 14.51% of the total effect, 95% Boot CI (−0.103, −0.008)]; (2) Perceived stress → Self-directed learning ability → Professional identity [effect value = −0.072, accounting for 18.66% of the total effect, 95% Boot CI (−0.106, −0.043)]; (3) Perceived stress → Self-control → Self-directed learning ability → Professional identity [effect value = −0.053, accounting for 13.73% of the total effect, 95% Boot CI (−0.079, −0.030)] (see Table 4; Figure 2).

**Table 4:** Total, direct, and indirect effects of perceived stress on professional identity.

**Paths**	**Effect**	**Boot SE**	**Bootstrap 95% (CI)**		**Relative mediation effect**
			**BootLL CI**	**BootUL CI**	
Total effect of X on Y	−0.386	0.033	−0.451	−0.321	
Direct effect of X on Y	−0.205	0.039	−0.281	−0.130	53.11%
Indirect effects 1 (X → M1 → Y)	−0.056	0.025	−0.104	−0.007	14.51%
Indirect effects 2 (X → M2 → Y)	−0.072	0.016	−0.106	−0.044	18.66%
Indirect effects 3 (X → M1 → M2 → Y)	−0.053	0.012	−0.078	−0.030	13.73%
Total indirect effect of X on Y	−0.181	0.027	−0.237	−0.128	46.89%

*N* = 675. Number of bootstrap samples for Bias-corrected bootstrap confidence intervals: 5,000. Level of confidence for all confidence intervals: 95%. X, perceived stress; M1, self-control; M2, self-directed learning ability; Y, professional identity.

## Discussion

This study investigated the influence of perceived stress on the professional identity among nursing students and the mediating role of self-control and self-directed learning ability in the relationship between perceived stress and professional identity. Specifically, the finding of this study show that: (1) Professional identity is negatively correlated with perceived stress, but positively correlated with self-control and self-directed learning ability among nursing students. (2) Self-control partially mediating role in the relationship between perceived stress and professional identity. (3) Self-directed learning ability partially mediating role in the relationship between perceived stress and professional identity. (4) Self-control and self-directed learning ability have a chain mediating role in the between stress perception and the professional identity among nursing students.

### Influence of perceived stress on professional identity

The results of this study found that perceived stress is a risk factor for professional identity. In other words, compared to nursing students with low perceived stress, those with high perceived stress have lower professional identity. This finding not only confirms hypothesis 1 of this study but also aligns with previous research results (5, 22, 39). Possible reasons include: firstly, due to a lack of practical experience, nursing students may struggle to handle various nursing tasks and emergencies during their internship, leading to a lack of self-confidence and a decrease in their identification with the nursing profession. Secondly, the high-pressure internship environment and challenging nature of the work may make it difficult for nursing students to adapt to the internship, leading to anxiety and a decrease in professional identity. Lastly, nursing students need to adapt and integrate into the healthcare team during their internship, which can lead to role transition conflicts. Role conflicts may cause interns to feel confused and uneasy, resulting in a decreased sense of identification with the nursing profession.

Furthermore, this result confirms the stress-adaptation model (12), which states that individuals with higher levels of perceived stress are more susceptible to emotional and cognitive disruptions, significantly affecting their adaptive capacity and hindering the development of professional identity. The negative impact of perceived stress on professional identity suggests the need to pay attention to the mental health of nursing students, provide appropriate psychological support and practical guidance, enhance their self-efficacy, help them adapt to the internship environment, alleviate perceived stress, and ultimately improve their sense of professional identity.

### Mediation through self-control

The results of this study found that self-control mediates the relationship between perceived stress and professional identity among nursing students, confirming hypothesis 2. This indicates that as the level of perceived stress increases, nursing students expend more self-control resources, resulting in lower professional identity. Previous research has consistently found that perceived stress negatively predicts self-control ability (22). Individuals with higher levels of perceived stress usually face more pressure and challenges, requiring them to expend more psychological and cognitive resources to cope with stress. Prolonged exposure to high levels of stress may result in a depletion of psychological resources and a decrease in self-control ability (39).

Research has found that self-control positively predicts professional identity among nursing students (12). Individuals with strong self-control abilities are better at regulating and managing their emotions, behaviors, and thoughts. They are able to control their emotions and behaviors more effectively to adapt and respond to job demands. This self-regulation ability helps individuals develop a positive work attitude and professional identity. Additionally, this study further validates social cognitive theory (10). According to social cognitive theory, an individual's self-control ability may influence their ways of coping with stress and its effectiveness. Individuals with stronger self-control abilities are more likely to adopt proactive coping strategies, thereby reducing the negative impact of stress. Perceived stress can affect professional identity by depleting self-control resources. This suggests that enhancing an individual's self-control ability can cultivate and improve professional identity among nursing students.

### Mediation through self-directed learning ability

The results of this study found that self-directed learning ability mediates the relationship between perceived stress and professional identity among nursing students, confirming hypothesis 2. This indicates that as the level of perceived stress increases, self-directed learning ability decreases, resulting in lower professional identity. Research has found that perceived stress negatively predicts self-directed learning ability, consistent with previous research (22). For nursing students, during the clinical internship stage, they often feel lack of confidence, confusion, communication difficulties, and unclear role positioning due to lack of practical experience, effective communication skills, and time management abilities, which may increase their stress levels (1, 4). It is well-known that moderate stress can promote positive learning and enhance self-directed learning ability, but excessive stress can lead to some problems that are not conducive to physical and mental health development, such as social anxiety, loneliness, learning fatigue, decreased professional self-efficacy, and smartphone addiction, resulting in a decrease in self-directed learning ability (10, 12, 39).

Research has found that self-directed learning ability positively predicts professional identity among nursing students (12, 13, 25). Nursing students with strong self-directed learning abilities are more likely to actively seek learning opportunities and challenges to continuously improve their knowledge and skills. Through self-directed learning, they are able to continuously grow and develop, enhancing their professional competence in the nursing field. The satisfaction and sense of achievement brought about by this self-growth and development contribute to strengthening nursing students' professional identity. Perceived stress can affect professional identity by reducing self-directed learning ability. This also suggests that improving professional identity can be achieved by reducing perceived stress and enhancing self-directed learning ability among nursing students.

### Mediation through self-control and self-directed learning ability

The results of this study found that perceived stress indirectly affects professional identity through the chain mediating effects of self-control and self-directed learning ability, confirming hypothesis 4. Research has found a positive correlation between self-control and self-directed learning ability, consistent with previous research (25, 40). There may be three possible reasons: Firstly, self-control refers to the ability of individuals to regulate and control their own thoughts, emotions, and behaviors. Nursing students with strong self-control are better able to self-regulate and manage their learning behaviors, set clear learning goals, plan learning time and strategies, and persist in execution. This self-regulation ability helps improve nursing students' self-directed learning ability. Secondly, nursing students with strong self-control are often able to establish effective learning habits, such as regularly scheduling study time, learning plans, and methods. These learning habits help improve learning efficiency and quality, enhancing nursing students' self-directed learning ability. Thirdly, nursing students with strong self-control are more capable of resisting external interference and temptation, able to concentrate their attention and energy on learning. They are better able to manage their time and energy, avoiding distractions from unrelated matters, thereby improving their self-directed learning ability.

Self-control and self-directed learning ability function as mediators in a chain relationship between perceived stress and professional identity among nursing students. This finding suggests that nursing students experiencing higher levels of perceived stress undergo a continuous depletion of self-control resources, which are inadequately replenished over time, ultimately leading to a decline in self-control ability. Individuals with lower self-control may demonstrate diminished self-restraint, face difficulties in developing effective learning habits, and struggle to resist external distractions and temptations, frequently resulting in lower self-directed learning ability, which hinders the establishment of a robust professional identity.

### Implication for theoretical and practice

The findings of this study may contribute to improving the professional identity of nursing students. On the one hand, these results theoretically underscore the potential mediating roles of self-control and self-directed learning in understanding the impact of perceived stress on professional identity. This research expands the understanding of how perceived stress influences the development of professional identity. Additionally, this study reinforces the stress-coping model by indicating that the detrimental effects of perceived stress on professional identity can be alleviated through the adjustment of self-regulation mechanisms, particularly by improving self-control and fostering self-directed learning skills. On the other hand, the results of this study have certain guiding significance for improving and enhancing the professional identity of nursing students. First, nursing educators should pay attention to students with high perceived stress, low self-control, and low self-directed learning ability. Second, emphasis should be placed on cultivating and improving nursing students' self-control and self-directed learning ability, guiding them to objectively evaluate stress-inducing events in the clinical internship from a positive perspective, thereby reducing the impact of perceived stress on professional identity and enhancing nursing students' professional identity. Third, well-known experts in the nursing field should be regularly invited to carry out a series of related lectures on career planning, so as to improve the recognition of nursing students on nursing career.

### Limitations and strengths

This study has certain limitations. First, 94.22% of the participants were female, and 5.78% were male. Future studies should include a larger proportion of male participants to obtain more comprehensive and representative results. Second, data were collected through online self-reporting, which may introduce recall and selection bias, potentially affecting data accuracy. Future research could incorporate face-to-face interviews or multiple data collection methods to enhance reliability. Third, the influence path of perceived stress on professional identity is multifaceted, and this study only focuses on the mediating role of self-control and self-directed learning ability, while not considering other influencing factors. Future studies should incorporate more relevant variables to construct a more comprehensive and systematic model. Fourth, this study used a cross-sectional design, limiting causal inferences. Although it provides valuable insights, it cannot definitively explain the causal relationship between perceived stress and professional identity. Future studies should consider longitudinal or experimental methods (e.g., aggregated crossover designs, multilevel linear models) to explore this relationship further. Lastly, convenience sampling may limit the representativeness of the sample. Future research should aim for random sampling to improve generalizability and external validity.

This study also has several strengths. The large sample size (*N* = 675) provides robust support for the study's conclusions and statistical power. The use of a large sample allows for more accurate estimation of effect sizes and enhances the credibility of the results. This study not only presents its results in a well-structured manner but also systematically explains the significance of the findings and their connection to existing theories, providing a solid foundation for future research. The study clearly reveals the complex relationships between perceived stress, self-control, self-directed learning ability, and professional identity among nursing students, and it is the first to validate this theoretical model in Chinese nursing students, making a substantial contribution to the theory and practice of nursing education. We encourage other researchers to conduct replication studies in diverse contexts and samples to validate our findings.

## Conclusion

The perceived stress directly affects professional identity among nursing students, but also indirectly influences it through self-control and self-directed learning ability. Research has found that self-control and self-directed learning ability positively predict professional identity, while perceived stress has a detrimental effect. Furthermore, this study found that for nursing students with high perceived stress, enhancing self-control and self-directed learning ability may be an effective way to improve professional identity. This study provides a theoretical basis and new perspectives for developing intervention programs to enhance nursing students' professional identity.

## Data Availability

The raw data supporting the conclusions of this article will be made available by the authors, without undue reservation.
